# Oyster reproduction is compromised by acidification experienced seasonally in coastal regions

**DOI:** 10.1038/s41598-017-13480-3

**Published:** 2017-10-16

**Authors:** Myrina Boulais, Kyle John Chenevert, Ashley Taylor Demey, Elizabeth S. Darrow, Madison Raine Robison, John Park Roberts, Aswani Volety

**Affiliations:** University of North Carolina Wilmington, Center for Marine Science, 5600 Marvin K. Moss Lane, Wilmington, NC 28409 USA

## Abstract

Atmospheric carbon dioxide concentrations have been rising during the past century, leading to ocean acidification (OA). Coastal and estuarine habitats experience annual pH variability that vastly exceeds the magnitude of long-term projections in open ocean regions. Eastern oyster (*Crassostrea virginica*) reproduction season coincides with periods of low pH occurrence in estuaries, thus we investigated effects of moderate (pH 7.5, *p*CO_2_ 2260 µatm) and severe OA (pH 7.1, *p*CO_2_ 5584 µatm; and 6.7, *p*CO_2_ 18480 µatm) on oyster gametogenesis, fertilization, and early larval development successes. Exposure at severe OA during gametogenesis caused disruption in oyster reproduction. Oogenesis appeared to be more sensitive compared to spermatogenesis. However, Eastern oyster reproduction was resilient to moderate OA projected for the near-future. In the context of projected climate change exacerbating seasonal acidification, OA of coastal habitats could represent a significant bottleneck for oyster reproduction which may have profound negative implications for coastal ecosystems reliant on this keystone species.

## Introduction

Atmospheric and oceanic carbon dioxide (CO_2_) concentrations have been rising during the past century due to anthropogenic CO_2_ emissions (combustion of fossil fuels). The oceans have absorbed nearly half of the anthropogenically-produced CO_2_ during the past century^1^, causing changes in marine carbonate chemistry and reduction in oceanic pH, also called ocean acidification (OA). Average open ocean pH is about 8.2 and current models predicting the degree of ocean acidification will reduce pH by 0.3 to 0.5 by the year 2100 and possibly by 0.8 to 1.4 by 2300, depending on the CO_2_ emission scenario^2,3^.

Although the open ocean generally adheres to these global projections, some coastal areas already experience severe acidification that exceed the magnitude of long-term projections for the open ocean. In estuaries, inter-annual pH fluctuates from 8.2 down to <7.0 on the east coast of the USA^4–8^ (Supplementary Table S1). These changes can persist for weeks, mainly occurring during spring and summer months. Carbonate chemistry changes are driven by a combination of environmental factors including higher temperature and biological productivity, and are further influenced by episodic weather events such large rainfall bringing nutrients via surface runoff and freshwater influx^5,7,9^. Additionally, some studies have demonstrated that coastal zones will not experience acidification gradually, as seen in the open ocean, but rather as increases in frequency, duration, and magnitude of acidification events^10,11^. Overall, OA may further exacerbate the natural seasonal pH fluctuations experienced by coastal habitats^12^.

Estuarine ecosystems are habitats for ecologically and economically important marine organisms. The sustainability of these populations relies on reproduction success (i.e. gametogenesis, spawning, fertilization, development of early-life stages), and juvenile and adult growth and survival. Reproduction is considered to be the most sensitive process to OA in many marine species^13,14^. Most studies on OA effects on reproduction have focused on larval development^15–19^, and our understanding of the potential impact of acidification on reproduction of marine organisms is limited by the scarcity of gametogenesis studies.

Though gametogenesis is a crucial phase for the success of reproduction, and therefore survival and evolution of species, studies investigating the effects of OA on gametogenesis have been only conducted in sea urchins. In the sea urchin, *Hemicentrotus pulcherrimus*, long-term effects of exposure to elevated *p*CO_2_ (*p*CO_2_ 919 μatm; pH 7.83, 9-month exposure) delayed gametogenesis and spawning by a month^20^. A decrease in the volume of sperm produced was indicated in the sea urchin, *Echinometra mathaei*, exposed to three elevated *p*CO_2_ for 6 weeks (pH 7.5-8.1; *p*CO_2_ ~485–1770 µatm)^21^; and gonad mass was found to be significantly reduced by severe acidification (i.e. higher than levels predicted for the year 2100; pH 7.19; 45-day exposure) in the green sea urchin (*Strongylocentrotus droebachiensis*)^22^. Studies on marine invertebrates have reported contrasting responses to moderate OA (i.e. levels predicted for the year 2100) on fertilization success, even for the same species. Byrne *et al*.^23–25^ and Guo *et al*.^26^ found that fertilization success is robust to moderately increased *p*CO_2_ in sea urchins (*Heliocidaris erythrogramma*, *Heliocidaris tuberculata*, *Tripneustes gratilla*, *Centrostephanus rodgersii*) and abalones (*Haliotis coccoradiata*, *Haliotis diversicolor* and *Haliotis discus hannai*). However, Graham *et al*.^27^ and Havenhand *et al*.^28^ observed a negative effect of near-future OA on fertilization success in the sea urchin *Paracentrotus lividus* and *Heliocidaris erythrogramma*. In the Sydney rock oyster (*Saccostrea glomerata*) and the Pacific oyster (*Crassostrea gigas*), Parker *et al*.^29,30^ and Barros *et al*.^31^ reported that fertilization success was reduced in moderately acidified seawater, whereas Kurihara *et al*.^32^ and Havenhand and Schlegel^33^ found no effect on fertilization for the Pacific oyster. The effects of severe OA have been rarely explored, but has been shown to have a negative effect in all species studied. It was found that fertilization success was reduced in abalones (*Haliotis diversicolor*, *Haliotis discus hannai*) and the Pacific oyster and Portuguese oyster (*Crassostrea angulata*) at pH values ≤ 7.43^26,31^.

The Eastern oyster (*Crassostrea virginica*) is a keystone species and the second most valuable bivalve fishery in the USA^34^. This species has been the focus of conservation and restoration efforts because oyster populations have declined worldwide^35,36^, and so have the ecosystem services they provide, including improved coastal water quality through filtration, and the creation of complex reefs that represent key habitat for numerous fish, invertebrate, and bird species^36–38^. In this species, a new gonad is formed seasonally during spring and summer months and gametes are released concurrently in both sexes, allowing external fertilization to occur in the water column^39^. The timing of Eastern oyster reproduction coincides with periods of low pH in estuaries. Though the success of reproduction is critical for sustainability of populations, the effect of OA on reproductive capacity (i.e. gametogenesis and fertilization success) have not been investigated in this species or any bivalve species. These data are crucial to understand the present (i.e. severe acidification currently experienced by coastal habitats) and future impacts of OA (i.e. moderate acidification projected for the open ocean) on important physiological processes for oysters and, to a larger extent, for marine populations.

In this study, we investigated how moderate and severe acidification affect reproduction in the Eastern oyster. Oysters were conditioned for 5 weeks under control, moderate OA predicted for the open ocean by the end of the century, and two severe OA treatments that coastal habitats are currently experiencing during the oyster reproductive season. Gametogenesis, fertilization, and D-larval development successes were assessed after the 5-week exposure period.

## Results

### Seawater chemistry parameters

Salinity was 35.5 ± 1.0 and 35.6 ± 0.3 PSU throughout the 5-week oyster conditioning and fertilization experiments, respectively. Mean seawater temperature during these two experiments was 21.2 ± 0.4 °C. Dissolved oxygen never decreased below 6.0 mg/L or 90% saturation. Values of pCO_2_ were 784 ± 89, 2260 ± 68, 5584 ± 277, 18480 ± 943 (mean ± SD) µatm in the pH 7.9, 7.5, 7.1, and 6.7 conditions, respectively. During the fertilization experiment, values of pCO_2_ were 803 ± 70 and 2239 ± 98 µatm in the pH 7.9 and 7.5 conditions, respectively. During the 48-h larval-development experiment, values of pCO_2_ were 798 ± 92 and 2276 ± 46 µatm in the pH 7.9 and 7.5 conditions, respectively. All seawater chemistry parameters are summarized in Supplementary Tables S2 and S3.

### Survival

Two oysters died during the 5-week experimental period in the pH 6.7 conditions. Survival was not significantly different among pH treatments.

### Gametogenesis stages and sex ratio

**Figure 1:**
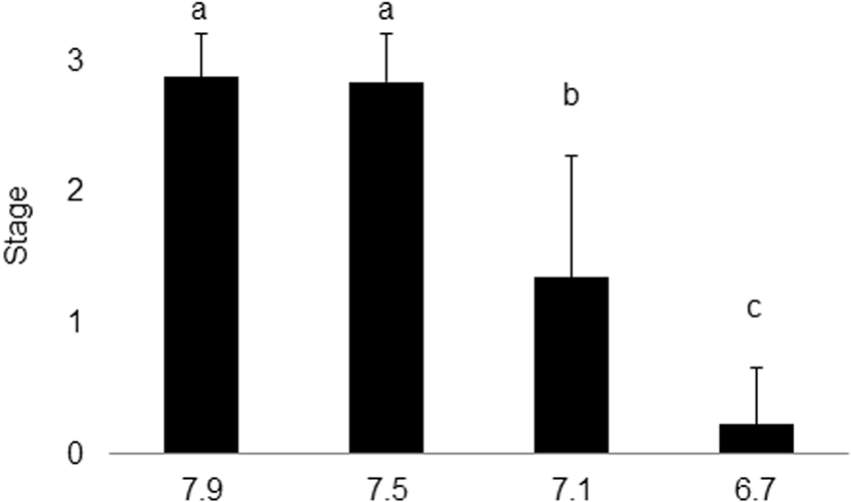
Mean oyster gametogenesis stages after 5-week conditioning at four pH levels. Stage 0: dormant phase, stage I: early development, stage II: late development, stage III: ripe gonad. Lower-case letters above bars indicate significant differences in means of gametogenesis stage among pH levels (Logistic regression, pH 7.9: n = 24, pH 7.5: n = 25, pH 7.1: n = 23, pH 6.7: n = 22 oysters; the hermaphrodite oyster was excluded from the statistical analysis, p < 0.05).

At day 1 of the exposure, histological examination revealed that all oysters were at stage 0 (no evidence of gonadal development) or I (early development of gametogenesis). At the end of the 5-week experimental period, pH significantly affected oyster gonadal development (p < 0.0001). Of the control group (pH 7.9), 21 oysters were in stage III (ripe gonad), and three oysters were in stage II (late development of gametogenesis, Supplementary Figure S1). Mean gametogenesis stage of individuals exposed at pH 7.9 was 2.9 ± 0.3 (mean ± SD) (Fig. 1). Oysters exposed at pH 7.9 and 7.5 displayed similar gametogenesis development (Fig. 1, Table 1, pH 7.5: 21 and 4 oysters in stage III and stage II, respectively; mean gametogenesis stage = 2.8 ± 0.4). However, oysters conditioned at pH 7.1 showed delayed gametogenesis compared to control (p < 0.0001), with 3 oysters at stage III, 6 oysters at stage II, 10 oysters at stage I, and 4 oysters at stage 0; and a mean gametogenesis stage of 1.3 ± 0.9 (Fig. 1, Table 1). Individuals exposed to pH 6.7 during gametogenesis underwent complete inhibition of gametogenesis, illustrated by the lack of gamete development (Fig. 1, Table 1, mean gametogenesis stage = 0.2 ± 0.4). These individuals were mostly in stage 0 (17 oysters) and the rest were in stage I (5 oysters).

**Table 1:** Chi-square tests performed on gametogenesis stages of oyster after 5-week conditioning at four pH levels.

	χ^2^	p
7.9 versus 7.5	0.12	0.73
7.9 versus 7.1	34.06	<0.0001
7.9 versus 6.7	63.68	<0.0001
7.5 versus 7.1	32.89	<0.0001
7.5 versus 6.7	64.96	<0.0001
7.1 versus 6.7	21.22	<0.0001

pH 7.9: n = 24, pH 7.5: n = 25, pH 7.1: n = 23, pH 6.7: n = 22 oysters; the hermaphrodite oyster was excluded from the statistical analysis.

Sex ratios (number of female: number of male) of oysters observed at the end of the conditioning are presented in Table 2 and were not different among pH 7.9, 7.5, and 7.1 (Table 3), nor different compared to the sex ratio observed at the beginning of the experiment (sex ratio: 1:0.5). However, sex ratio of oysters exposed at pH 6.7 was significantly different compared to pH 7.9, 7.5, and 7.1 (Table 3). One hermaphrodite individual was found at pH 7.1 at the end of conditioning.

**Table 2:** Sex ratio of oysters after 5-week conditioning at four pH levels.

pH	Number					Sex ratio (F:M)
	Total	Female	Male	Hermaphrodite	Undetermined	
7.9	24	15	9	0	0	1:0.6
7.5	25	14	11	0	0	1:0.8
7.1	24	8	11	1	4	1:1.4
6.7	22	0	5	0	17	0:5

F: female, M: male.

**Table 3:** Chi-square tests performed on sex ratio of oyster after 5-week conditioning at four pH levels.

	χ^2^	p
7.9 versus 7.5	0.21	0.64
7.9 versus 7.1	1.78	0.18
7.9 versus 6.7	8.41	0.00
7.5 versus 7.1	0.84	0.36
7.5 versus 6.7	7.16	0.01
7.1 versus 6.7	4.69	0.03

pH 7.9: n = 24, pH 7.5: n = 25, pH 7.1: n = 19, pH 6.7: n = 5 oysters; the hermaphrodite oyster and the oysters at stage 0 were excluded from the statistical analysis.

**Figure 2:**
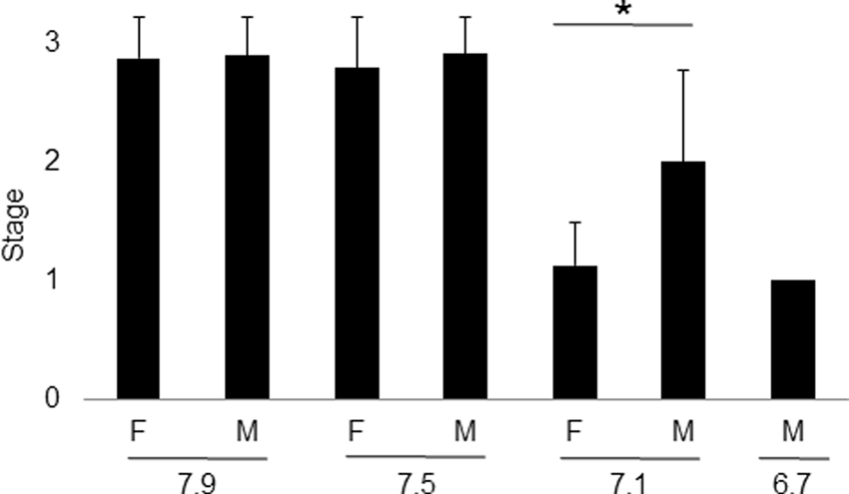
Mean gametogenesis stages of female and male oysters after 5-week conditioning at four pH levels. F: female, M: male, stage I: early development, stage II: late development, stage III: ripe gonad. Asterisk above bars indicates significant difference in gametogenesis stages between gender (Logistic regression, pH 7.9: n = 24, pH 7.5: n = 25, pH 7.1: n = 19, pH 6.7: n = 5; the hermaphrodite oyster, the oysters at stage 0, and those exposed at pH 6.7 (lack of females) were excluded from the statistical analysis; p < 0.05).

No difference between female and male gamete development was found in individuals exposed at pH 7.9 and pH 7.5 (Fig. 2, Table 4). However, oogenesis was more sensitive to severe OA than spermatogenesis, as shown by significant differences in mean gametogenesis stage between females and males at pH 7.1 (Fig. 2, Table 4, mean gametogenesis stage of females and males were 1.1 ± 0.4 and 2.0 ± 0.8, respectively), and the complete lack of females in oysters conditioned at pH 6.7. Females were identified in stages I (7 oysters) and II (1 oyster), whereas some males developed up to stage III (3 oysters) during the 5-week conditioning at pH 7.1 Mean gametogenesis stage of males conditioned at pH 6.7 was 1.0 ± 0.0.

**Table 4:** Chi-square tests performed on gametogenesis stages of female and male oysters after 5-week conditioning at four pH levels.

	χ^2^	p
pH 7.9 - F versus M	0.03	0.87
pH 7.5 - F versus M	0.73	0.39
pH 7.1 - F versus M	8.24	0.02

F: female, M: male. pH 7.9: n = 24, pH 7.5: n = 25, pH 7.1: n = 19; the hermaphrodite oyster, the oysters at stage 0, and those exposed at pH 6.7 (lack of females) were excluded from the statistical analysis.

### Fertilization and early larval development successes

Procedures to assess the fertilization and D-larval development successes of oysters after the 5-week experimental period were only conducted in oysters conditioned at pH 7.9 and 7.5 because no ripe gonads were found in females in pH 7.1 treatment and no females were observed at pH 6.7. Fertilization success of gametes was 80 ± 5% and 74 ± 4% at pH levels 7.9 and 7.5, respectively, and was not reduced by low pH (pH 7.5/*p*CO_2_ 2239 ± 98 µatm) compared to control (pH 7.9/*p*CO_2_ 803 ± 70 µatm). Similar results were found for early larval development. D-larval yield was 78 ± 3% and 70 ± 6% at pH levels 7.9 and 7.5, respectively. Shell lengths were not significantly different between D-larvae reared at pH 7.5 (79.3 ± 4.8 µm) and pH 7.9 (82.6 ± 3.4 µm).

## Discussion

This experiment was designed to assess the responses of Eastern oyster (*Crassostrea virginica*) reproductive capacity to a moderate level of OA predicted for the open ocean by the end of the century, and to two severe OA levels that coastal habitats are currently experiencing during the oyster reproductive season.

Moderate acidification (pH 7.5, *p*CO_2_ 2260 µatm) did not impact Eastern oyster gametogenesis, fertilization success, or early development of their offspring (up to the D-larval stage, 48 h after fertilization) demonstrating the resilience Eastern oyster reproduction exhibits in response to OA projected for the near-future. Severe acidification (pH 7.1 and 6.7/*p*CO_2_ 5584 and 18480 µatm), however, delayed and inhibited gametogenesis. To our knowledge, this is the first assessment of the impacts of OA on gametogenesis success of oysters, and literature addressing this question in marine invertebrates is limited to sea urchins. In sea urchins, gametogenesis was found to be highly sensitive to near-future OA^20,21^.

Our results highlight a threshold in pH tolerance of reproduction below which gonadal development and fertilization success are adversely altered in the Eastern oyster. Fertilization capacity was tested for a wide range of pH levels in the Pacific oyster. Studies indicate that fertilization success is robust to OA projected for the near-future, but highly sensitive to higher acidification (pH < 7.4) in this species. Kurihara *et al*.^32^ and Havenhand and Schlegel^33^ found no effect of moderate OA (pH 7.42 and pH 7.8, respectively) on fertilization in the Pacific oyster. Barros *et al*.^31^ confirmed this finding and also observed a decrease of 50% of the fertilization rate in pH 7.37 (3,573 µatm *p*CO_2_) compared to control (pH 8.09). Similarly, Byrne *et al*.^23^ compared data on sea urchins and highlighted that their fertilization was only affected by pH < 7.4. Our study shows that in the Eastern oyster, it is likely that the threshold in pH tolerance of reproduction is set around pH 7.4 as in the Pacific oyster and sea urchins, but this remains to be tested at a finer pH range between 7.5 and 7.1.

Effects of OA on the physiology of oysters are still poorly understood^40^. It has been demonstrated that in response to elevated *p*CO_2_, oxidative stress and basal metabolic costs are increased in the Eastern oyster^40,41^. Higher costs of basal metabolism were suggested to be additional energy needs for acid-base regulation and biomineralization-related enzymes^40^. However, these higher energy needs can probably not be compensated by increasing feeding activity at very low pH levels. Feeding has been found to be suppressed at pH 7.0 in the Pacific oyster and reduced in the European flat oyster, *Ostrea edulis*, at pH 7.2^42^. The total energy of an organism must be allocated in such a way that balances maintenance, growth, and reproduction, and under low energy input or stressful conditions, energy is allocated to maintenance rather than growth and reproduction^43,44^. In the Pacific oyster, progress of gametogenesis was found to be delayed during starvation^45^. Increased energy demand led to reduced gonadal growth in the green sea urchin exposed for 45 days to severe *p*CO_2_ (pH 7.19)^22^. Furthermore, in poor and stressful environmental conditions, such as pollution, it has been observed that fewer oysters differentiate as females during gametogenesis^46,47^ because oogenesis is a higher-energy cost process than spermatogenesis^48,49^. The female gamete needs to accumulate nutrient reserves (protein, lipid, carbohydrates, mRNA) required for embryo and early larval development^50,51^. Gametogenesis of the Eastern oyster depends on energy available for oocyte and sperm production. According to our results, the Eastern oyster seems to tolerate or compensate for the higher energy cost and stress induced by living in a moderately acidified environment (pH 7.5) with respect to reproduction. However, the higher energy needs induced by severe OA (pH 7.1 and 6.7) probably resulted in less energy available for reproduction. This could have contributed to delay and inhibition of gamete development observed in our study. Higher sensitivity of oogenesis to elevated *p*CO_2_ compared to spermatogenesis is likely to reflect the greater energy cost of producing oocytes compared to spermatogenesis.

In our work, we found inter-individual variability among males in the responses of gametogenesis to OA, with some individuals developing and maturing spermatozoa under severe acidification (pH 7.1). Inter-individual variability in the capacity to tolerate or compensate for elevated *p*CO_2_ could be attributed to genotypes that were more tolerant to the acidified environment. In coastal zones, the Eastern oyster is already subjected to seasonal severe acidification of their habitats during their reproductive season. These fluctuations may have foster genetic variability with respect to acidification resistance in these populations of oysters^18,52,53^. Our findings reveal a potential for selective adaptation to severe OA in male individuals of the Eastern oyster. Whether females will have this capacity to adapt to severe elevations in *p*CO_2_ remains unclear. Parker *et al*.^54,55^ found that exposure of adult Sydney rock oysters had positive carry-over effects on their offspring, including improved capacity to regulate extracellular pH at elevated CO_2_. These carry-over effects originating during parental exposure will likely play an important role in oysters’ adaptive capacity for acclimation to long-term ocean acidification.

The timing of gametogenesis and then spawning generally converge to match the most suitable environmental conditions for the survival of the offspring^56^. In the context of projected climate change exacerbating seasonal acidification and increasing frequency, duration, and magnitude of storm events; severe OA could become a serious threat to oyster reproduction, especially for oogenesis. Asynchronous gametogenesis between sexes or delayed gametogenesis during the natural reproductive season would probably result in reduced fertilization success and fitness of larvae, and alter recruitment dynamics with respect to phenology. These changes could lead to increased post-settlement mortality, already hypothesized to limit the expansion of adult natural oyster reefs in the southeast U.S.A.^57^. Oyster reefs are crucial to ecosystems and their services, and restoration of oyster reef is a priority for habitat management, recovery, and conservation^36^. OA of some coastal habitats should be considered in future oyster restoration efforts, especially as timing of seasonal low pH events correspond with periods of oyster recruitment. Finally, complete failure of gametogenesis in females would represent a significant bottleneck in Eastern oyster reproduction, which could lead to decreased physiological function and growth rates^58^.

In conclusion, in the context of projected climate change, severe OA (pH 7.1, *p*CO_2_ 5584 µatm; and 6.7, *p*CO_2_ 18480 µatm) of coastal habitats could become a serious threat to oyster reproduction, especially for oogenesis. This would result in potential severe consequences for natural oyster populations and aquaculture. However, predicted changes in OA for the next century (pH 7.5, *p*CO_2_ 2260 µatm) do not represent a major threat for the gametogenesis, fertilization success, and early larval development of the Eastern oyster. Our work highlights the need for studies investigating a broader range of OA for marine species living in coastal areas that are already exposed to acidified environment. Furthermore, most OA studies have focused on development of early-life stages, however, for all sensitive species, impacts of OA on reproduction in its entirety must be considered, including not only assessment of fertilization success, but also gametogenesis, for understanding of links between climate change and evolutionary processes. Finally, the intra-individual variability in the response of reproduction to elevated *p*CO_2_ suggest a potential for selective adaptation in male individuals of the Eastern oyster. Improved understanding of the impact of ocean acidification on oyster reproduction and their adaptive capacity requires transgenerational studies investigating underlying mechanisms for acclimation and adaptation.

## Method

### Animal maintenance and experimental exposures

Oysters from the Shellfish Research Hatchery of the University of North Carolina Wilmington were reared in cages in the Intracoastal Waterway (GPS 34°8′25.4″ N, 77°51′44.5″ W) for 2 years and collected in March 2016 (n = 117; total weight = 38.1 ± 14.3 g, mean ± SD). The oysters were divided into 8 batches (n = 14 to 15 oysters per batch, depending on oyster weight), and batches were randomly assigned to one of 8-30 L tanks.

Oysters were acclimated for one week. During the acclimation and following exposure periods, seawater was pumped directly from the nearby estuary, filtered through a sand filter, filtered at 1 µm and UV-sterilized, 21 °C (FSW). With continuous bubbling of ambient air, all tanks remained saturated with respect to dissolved oxygen (~7 mg/L). Water was circulated within each tank using submersible pumps (SP-800, Resun). To act as biological filters, a bag containing Bio Balls Filter Media and ceramic rings (Fluval Biomax Bio Rings Filter Media) was placed in each tank. Each tank (closed system) received daily a one-third by volume water change, and a full water change every two or three days if ammonia and nitrate levels (saltwater test kit API, daily measurements) reached 0.25 and 5 ppm respectively. However, no tank needed to be changed more often than others. During water changes and readjustments of seawater pH in tanks, oysters were removed out of the tanks (30 minutes maximum) and faecal matter was removed from the tanks using an aquarium vacuum pump. Each tank was checked daily for any dead oyster which was then removed from the tank. Oysters were fed daily *ad libitum* with a mix of fresh algae (*Tisochrysis lutea, Chaetoceros muelleri*, *Thalassiosira weissflogii*, *Tetraselmis* sp., *Rhodomonas salina*, *Pavlova lutheri*, 2 × 10^9^ cells/day/oyster) provided by the Shellfish Research Hatchery.

During the acclimation period, seawater pH was ambient to the local estuary (7.84 ± 0.17). After the acclimation period, oysters were conditioned at 4 levels of lowered pH for 5 weeks (pH 7.9, 7.5, 7.1 and 6.7; 2 tanks/pH level). Though average open ocean pH is around 8.2, pH 7.9 was determined to be the ambient estuarine pH using pH monitoring data for the local estuary^8^ and thus was used as the control pH condition during our study. pH 7.5 was chosen to reflect the near-future level of ocean acidification (moderate acidification), as current scenarios predict a reduction in pH by 0.3 to 0.5 by 2100^2,3^. The two lower pH levels (i.e. 7.1, 6.7) were chosen to represent severe acidification conditions estuaries already experienced during spring and summer months, coinciding with the reproductive season of the Eastern oyster.

Acidification was achieved by manipulation of pH by direct aeration of the seawater with air enriched with pure gaseous CO_2_ controlled by independent pH negative feedback systems^55^. Briefly, each system consisted of a pH probe, pH controller (MC122, Milwaukee), and solenoid valve (MA957, Milwaukee). When pH rose above the desired level, the solenoid valve would open and dose the seawater with air enriched with CO_2_. Target pH values were determined using NBS scale (pH_NBS_, pH meter Thermo Scientific Orion 4 star calibrated with Metrohm buffer solutions in single use sachets prior to each use) and the achieved pH levels were then used to set the respective pH controllers.

### Seawater chemistry parameters

Seawater parameters were measured daily in each tank. Temperature, salinity, and dissolved oxygen concentrations were recorded using a YSI multiparameter probe (Professional Plus). Seawater pH_NBS_ was measured using a pH meter (Thermo Scientific Orion 4 star). For total alkalinity (A_T_) assays, seawater samples were collected daily in air-tight 20 mL vials without air space to prevent gas exchange with the atmosphere, stabilized by mercuric chloride poisoning^59^. Samples for alkalinity assays were collected while other seawater chemistry parameters were measured. Alkalinity samples were kept in the dark and analyzed within three months of collection. Seawater alkalinity was determined with an automatic titrator (Metrohm 848 Titrino Plus) and Dickson seawater (Batch 144) according to Dickson *et al*.^59^. Other carbonate system parameters were calculated from temperature, salinity, A_T_, and pH_NBS_ using the CO2SYS program (http://cdiac.ornl.gov/ftp/co2sys/) with dissociation constants (K1 and K2) according to Mehrback *et al*.^60^ refit by Dickson and Millero^61^, and KHSO_4_ dissociation constant after Dickson^62^.

### Histological analysis

Following the acclimation period, 20 oysters were randomly selected from the 8 tanks (n = 2 to 3 per tank depending on oyster weight) to determine both their sex and gametogenesis stage at the beginning of the experiment by qualitative histology. These two parameters were also assessed for all oysters following the 5-week experimental period (n = 95). Preparation for qualitative histology was adapted from Volety *et al*.^63^. Briefly, each individual oyster was shucked and a 3-mm transverse section of the visceral mass was excised on each oyster just anterior to the labial palps. Each tissue sample was placed in a cassette, immediately fixed for histology in Davidson’s solution and kept at 4 °C for a week, before being switched to 70% ethanol overnight. The tissue samples were then dehydrated in ascending ethanol solutions, cleared with xylene, then embedded in paraffin wax (MICROM STP 120, Thermo Scientific; TEC^TM^ II tissue embedding center, General data) before 7-μm thick sections were cut using a rotary microtome (MICROM HM 325, Thermo Scientific). Each tissue section was then mounted on a slide, and stained with Harry’s hematoxylin-Eosin Y. Tissue sections were examined under a light microscope (Olympus BX41) to determine the sex and gametogenesis stage of each oyster according to the reproductive scale reported by Steele and Mulcahy^64^. Briefly, the gametogenesis stage 0 defines the dormant phase of gametogenesis with no evidence of gonadal development. Stage I corresponds to the early development of gametogenesis, with gonadal tubules filled with spermatogonia and spermatocytes in males, or follicles filled with oogonia in females, but much of the gonad is still connective tissue. Stage II is defined as the late development of gametogenesis, gametes are still maturing. Tubules are filled with spermatids and spermatozoa and follicles filled with vitellogenic oocytes still attached to the follicle walls. Finally, stage III is characterized as the ripe gonad ready for spawning, tubules and follicles are filled with mature spermatozoa with flagella or mature oocytes unattached to the follicle walls, respectively.

### Collection of gametes and success of fertilization and early larval development

Fertilization and D-larval development successes were investigated only in ripe oysters (i.e. stage III) to avoid any effect of gametogenesis stage on these parameters. Stages of oysters were later confirmed by qualitative histology. Oocytes and sperm were collected for each individual by stripping gonads^65,66^. Briefly, for each oyster, gametes were collected in 10 mL of FSW at 21 °C and at the same pH level that the adults were conditioned at (FSW_pH_). Oocytes were sieved through 100 µm, concentrated on 20 µm mesh and transferred into a 2-L graduated cylinder filled with FSW_pH_. Sperm was sieved through 60 µm mesh. For each oyster, a 1-mL sample of gametes was diluted to 1:1000 in FSW to determine gamete concentration by flow cytometry (duplicate, EasyCyte Plus cytometer, Guava Millipore). Concentration of gametes were adjusted to 50,000 oocytes/mL and 10^7^ spermatozoa/mL by further dilution in FSW_pH_.

For each tank, a pool of oocytes from 3 females were fertilized with a pool of spermatozoa from 3 males from the same tank according to Boulais *et al*.^66^. Briefly, six replicate batches of 25,000 oocytes were fertilized in 50 mL FSW_pH_. After 30 minutes of contact between spermatozoa and oocytes, beakers were filled up to 1.8 L with FSW_pH_.

To estimate the fertilization success (number of trochophores/number of oocytes), the number of trochophore larvae was assessed 24 hours after fertilization: each of three individual beakers was sieved on a 40 µm mesh and concentrated in a 10-mL graduated cylinder. Trochophore larvae were enumerated by microscopic counts (3 × 50 µL), adding one drop of 4% formaldehyde solution to prevent their movement.

The success of early larval development (number of D-larvae/number of oocytes) was assessed 48 hours after fertilization in the three remaining beakers following the same methodology as measurement of fertilization success. To describe D-larval shell length, diameters of 50 D-larvae were analysed using a camera Olympus DP73 connected to a microscope (Olympus BX41). D-larvae diameters were measured using Image J software (n = 150 larvae for each beaker).

Seawater chemistry parameters were assessed immediately after filling the beakers with FSW_pH_ and before estimating the fertilization and D-larval development successes; 24 hours and 48 hours after fertilization, respectively.

### Statistical analyses

Comparison for gametogenesis stages (0, I, II, or III) and sex ratio differences among pH levels were determined using logistic regression. Prior to statistical analyses, results of oyster gametogenesis stages were pooled for each pH level to analyze one distribution per pH condition (individuals = replicates), after verifying there were no statistical differences between tanks in seawater chemistry parameters using Student’s t-test; and gametogenesis stages and sex ratio using Fisher’s exact test. One hermaphrodite oyster was excluded from the statistical analysis of gametogenesis stages (gametogenesis stage not determined) and sex ratios, and oysters at stage 0 (sex not determined) were also excluded from the sex ratio analysis. Percentage data (survival, trochophore-larval and D-larval yields) were arcsin square-root transformed prior to statistical analyses. Differences in survival were assessed using Kruskal-Wallis H test. Trochophore-larval and D-larval yields were compared using a Student’s t-test (pH 7.9 versus pH 7.5). A value of p < 0.05 was considered as significant. Statistical analyses were performed using JMP software.

### Data Availability

The datasets analyzed during the current study are available from the corresponding author on reasonable request.

## Electronic supplementary material

Supplementary information
