# Supplementary material for: Oyster reproduction is compromised by acidification experienced seasonally in coastal regions
Source: Sci Rep. 2017 Oct 16;7:13276. doi: 10.1038/s41598-017-13480-3 (PMC5643346; doi:10.1038/s41598-017-13480-3)
Supplement: Supplementary file 1 — Supplementary information [file 41598_2017_13480_MOESM1_ESM.pdf]

Supplementary Information

Oyster reproduction is compromised by acidification experienced seasonally in coastal regions

Myrina Boulais*, Kyle John Chenevert, Ashley Taylor Demey, Elizabeth S. Darrow, Madison Raine Robison, John Park Roberts, Aswani Volety.

University of North Carolina Wilmington, Center for Marine Science, 5600 Marvin K. Moss Lane, Wilmington, NC 28409, USA.

* email: myrina.boulais@gmail.com

Supplementary figure S1

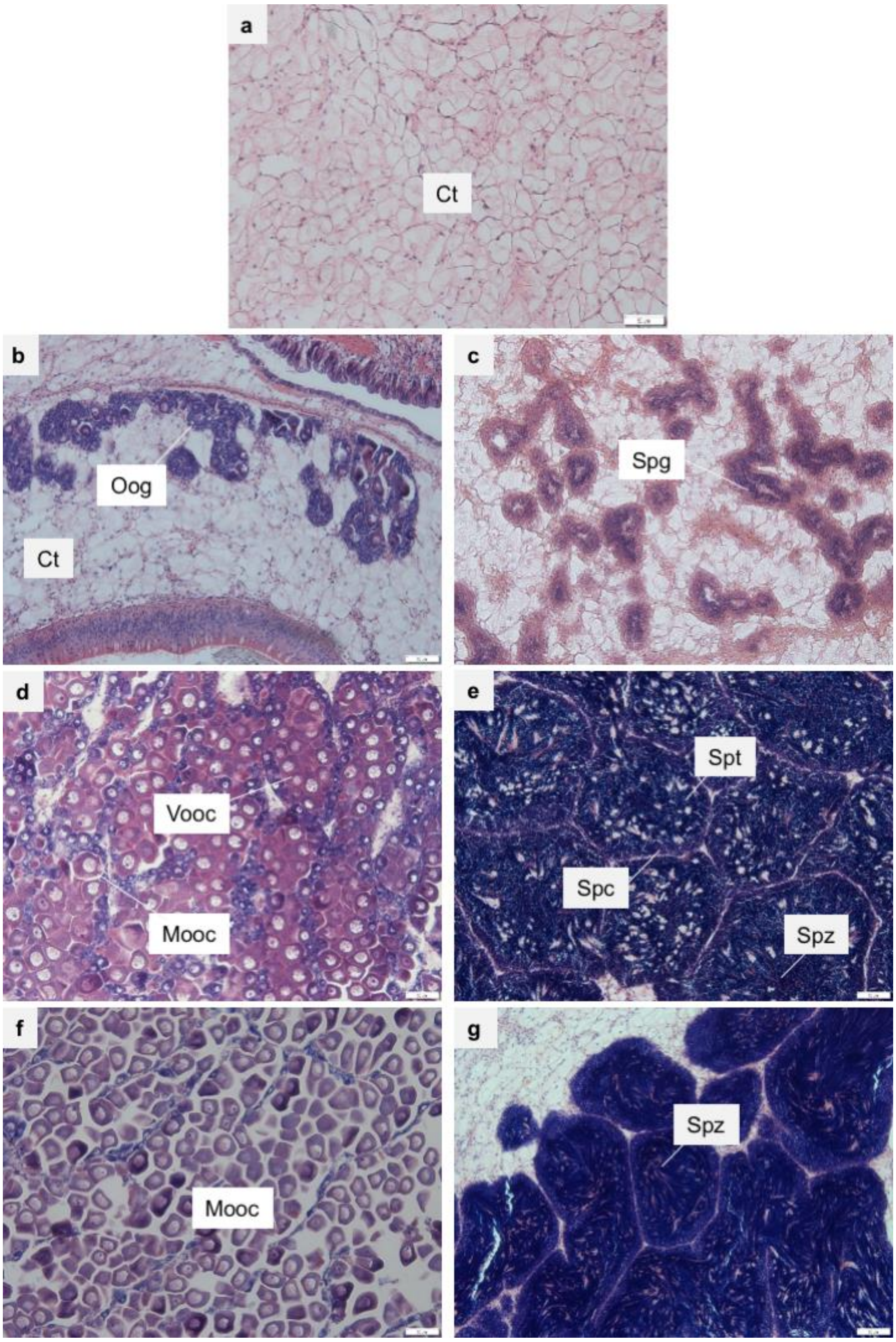

Supplementary Figure 1. Histological description of the different gametogenesis stages of the Eastern oyster observed at the end of the four pH level conditioning. (a) gonad illustrating the dormant phase, observed in the pH 6.7 condition. Gonads of **(b)** a female and **(c)** a male illustrating early development (Stage I) observed at pH 7.1 or 6.7. A female **(d)** and a male **(e)** gonad illustrating late development (Stage II) observed at pH 7.9, 7.5, and 7.1. A female **(f)** and a male **(g)** ripe gonad (Stage III) observed at pH 7.9, and 7.5. Ct: conjunctive tissue, Oog: oogonia, Vooc: vitellogenic oocyte, Mooc: mature oocyte, Spg: spermatogonia, Spc: spermatocytes, Spt: spermatids, Spz: spermatozoa. Scale bar = 50 µm.

Supplementary Table S1, S2, and S3

Supplementary Table 1. Summary of interannual pH variability in East Coast, USA estuaries. NA, Not Available.

Type of measurement	pH	Site	Reference
Average (minimum)	7.2 - 7.8 (6.9 - 7.6)<br>depending on sites, in summer	Charleston Harbor, SC	1
ΔpH	0.3<br>minimum in pH in the summer	Pivers Island Coastal Observatory, Beaufort, NC<br>34.7181° N, 76.6707 ° W	2
Average (minimum)	8.2 (8.1) at the end of winter<br>7.6 (7.2) in late summer	Flax Pond, Long Island, NY<br>40°57.78’ N, 73°8.22’ W	3
Average (minimum)	NA (7.9) in winter<br><7.9 (<7.0) in summer	Western Long Island Sound, NY-CT<br>41.11° N, 72.86° W	4

Supplementary Table 2. Water chemistry parameters during the 5-week oyster conditioning (4 pH levels) and fertilization (2 pH levels) experiments (mean ± SD). NBS, National Bureau of Standards; *p*CO2, partial pressure of CO_2; DIC, total dissolved inorganic carbon; SW, seawater; Ω, saturation state.

Parameter	Oyster conditioning experiment			
pH_{NBS}	7.90 ± 0.06	7.48 ± 0.03	7.12 ± 0.01	6.71 ± 0.02
pCO_2 (µatm)	784 ± 89	2260 ± 68	5584 ± 277	18480 ± 943
DIC (µmol kg^{-1} SW)	2048 ± 68	2215± 83	2476 ± 114	3499 ± 78
CO_3^{2-} (µmol kg^{-1} SW)	109 ± 15	45 ± 5	21 ± 1	11 ± 0.32
HCO_3^- (µmol kg^{-1} SW)	1915 ± 57	2099 ± 80	2280 ± 106	2911 ± 57
$\Omega_{Calcite}$	2.59 ± 0.37	1.07 ± 0.11	0.51 ± 0.03	0.25 ± 0.01
$\Omega_{Aragonite}$	1.69 ± 0.24	0.70 ± 0.08	0.33 ± 0.02	0.16 ± 0.01
Total alkalinity (µmol kg^{-1} SW)	2185 ± 87	2211 ± 90	2332 ± 108	2936 ± 58

Supplementary Table 3. Water chemistry parameters (mean ± SD) during the fertilization and larval development experiments (2 pH levels). NBS, National Bureau of Standards; *p*CO2, partial pressure of CO_2; DIC, total dissolved inorganic carbon; SW, seawater; Ω, saturation state.

Parameter	Fertilization experiment		Larval development experiment	
pH_{NBS}	7.90 ± 0.04	7.49 ± 0.02	7.90 ± 0.03	7.48 ± 0.02
pCO_2 (µatm)	803 ± 70	2239 ± 98	798 ± 92	2276 ± 46
DIC (µmol kg^{-1} SW)	2094 ± 18	2272 ± 12	2070 ± 73	2250 ± 43
CO_3^{2-} (µmol kg^{-1} SW)	108 ± 9	48 ± 3	108 ± 6	45 ± 1
HCO_3^- (µmol kg^{-1} SW)	1961 ± 11	2154 ± 11	1937 ± 75	2133 ± 41
$\Omega_{Calcite}$	2.56 ± 0.21	1.15 ± 0.08	2.58 ± 0.13	1.08 ± 0.02
$\Omega_{Aragonite}$	1.67 ± 0.13	0.75 ± 0.05	1.68 ± 0.09	0.70 ± 0.01
Total alkalinity (µmol kg^{-1} SW)	2228 ± 33	2273 ± 2	2205 ± 60	2244 ± 43

Chemistry parameter values for 7.9 and 7.5 conditions were in the range of those reported in previous studies conducted on the Eastern oyster on the East coast of the USA[5,6].

Supplementary references

1. Ringwood, A. H. & Keppler, C. J. Water quality variation and clam growth: Is pH really a non-issue in estuaries? *Estuaries* **25**, 901–907 (2002).

2. Johnson, Z. I. *et al.* Dramatic Variability of the Carbonate System at a Temperate Coastal Ocean Site (Beaufort, North Carolina, USA) Is Regulated by Physical and Biogeochemical Processes on Multiple Timescales. *PLOS ONE* **8**, e85117 (2013).

3. Baumann, H., Wallace, R. B., Tagliaferri, T. & Gobler C.J. Large Natural pH, CO2 and O2 Fluctuations in a Temperate Tidal Salt Marsh on Diel, Seasonal, and Interannual Time Scales. *Estuaries Coasts* **38**, 220–231 (2015).

4. Wallace, R. B., Baumann, H., Grear, J. S., Aller, R. C. & Gobler, C. J. Coastal ocean acidification: The other eutrophication problem. *Estuar. Coast. Shelf Sci.* **148**, 1–13 (2014).

5. Beniash, E., Ivanina, A., Lieb, N.S., Kurochkin, I. & Sokolova, I.M. Elevated level of carbon dioxide affects metabolism and shell formation in oysters *Crassostrea virginica*. *Mar. Ecol. Prog. Ser.* **419**, 95–108 (2010).

6. Gobler, C. J. & Talmage, S. C. Physiological response and resilience of early life-stage Eastern oysters (*Crassostrea virginica*) to past, present and future ocean acidification. *Conserv. Physiol.* **2**, cou004 (2014).
